# Substance use disorders and COVID-19: reflections on international research and practice changes during the “poly-crisis”

**DOI:** 10.3389/fpubh.2023.1201967

**Published:** 2023-07-17

**Authors:** Hannah Carver, Teodora Ciolompea, Anna Conway, Carolin Kilian, Rebecca McDonald, Andia Meksi, Marcin Wojnar

**Affiliations:** ^1^Faculty of Social Sciences, University of Stirling, Stirling, United Kingdom; ^2^Drug Addiction Evaluation and Treatment Center, Saint Stelian, Bucharest, Romania; ^3^The Kirby Institute, University of New South Wales, Sydney, NSW, Australia; ^4^Centre for Addiction and Mental Health, Institute for Mental Health Policy Research, Toronto, ON, Canada; ^5^Norwegian Centre for Addiction Research (SERAF), Institute of Clinical Medicine, Oslo University, Oslo, Norway; ^6^National Institute of Public Health, Tirana, Albania; ^7^Department of Psychiatry, Medical University of Warsaw, Warsaw, Poland; ^8^Department of Psychiatry, University of Michigan, Ann Arbor, MI, United States

**Keywords:** drugs, alcohol, pandemic, addictions, treatment service, opioids, harm reduction

## Abstract

Since March 2020, the COVID-19 pandemic has had a disproportionately high toll on vulnerable populations, coinciding with increased prevalence of alcohol-and drug-related deaths and pre-existing societal issues such as rising income inequality and homelessness. This poly-crisis has posed unique challenges to service delivery for people with substance use disorders, and innovative approaches have emerged. In this Perspectives paper we reflect on the poly-crisis and the changes to research and practice for those experiencing substance use disorders, following work undertaken as part of the InterGLAM project (part of the 2022. Lisbon Addictions conference). The authors, who were part of an InterGLAM working group, identified a range of creative and novel responses by gathering information from conference attendees about COVID-19-related changes to substance use disorder treatment in their countries. In this paper we describe these responses across a range of countries, focusing on changes to telehealth, provision of medications for opioid use disorder and alcohol harm reduction, as well as changes to how research was conducted. Implications include better equity in access to technology and secure data systems; increased prescribed safer supply in countries where this currently does not exist; flexible provision of medication for opioid use disorder; scale up of alcohol harm reduction for people with alcohol use disorders; greater involvement of people with lived/living experience in research; and additional support for research in low- and middle-income countries. The COVID-19 pandemic has changed the addictions field and there are lessons for ongoing and emerging crises.

## Introduction

From March 2020 onwards, drug and alcohol treatment services around the world had to swiftly revise operating procedures to attend to the acute pressures of COVID-19 infection control and ever-changing social distancing requirements while ensuring continuity of care (1–3). For those experiencing substance use disorders and frontline workers alike, the pandemic added fuel to the fire of pre-existing overlapping crises of budget cuts, poverty, homelessness, and worsening health outcomes (including drug- and alcohol-related deaths), effectively creating a “poly-crisis.” However, across the addictions field, the COVID-19 pandemic also opened a “policy window of opportunity” to trial and evaluate new interventions or make bold changes to practice that were previously considered unthinkable or unfeasible (3, 4).

On 23^rd^-25^th^ November 2022, the 4^th^ European Conference on Addictive Behaviors and Dependence (also: “Lisbon Addictions 2022,” “LxAddictions22: Global addictions”) was convened in Portugal, offering–for the first time since the start of the pandemic–a forum for multidisciplinary networking and exchange of COVID-19-related experiences and evidence-based practices. As part of the InterGLAM (“Global perspectives on addictions and drug markets”; see Funding) thematic strand of the conference, our working group was established in June 2022 with the task of exploring “the impact of the global public health crisis in the addictions field” (5). The group was one of five convened by the project, each working on a different theme related to addiction. Co-facilitated by HC and TC, our group comprised academics and clinicians working in Albania, Australia, Canada, Greece, Norway, Poland, Romania, South Africa, and the United Kingdom. Participation in the working group provided an opportunity to understand the ongoing issues resulting from COVID-19 and for unpublished work to be shared internationally.

To capture a wider range of experiences, we emailed an online survey to the participants of the other four InterGLAM working groups in the lead-up to the conference (7 November 2022). During and after the conference, we also emailed the survey to all conference attendees who presented COVID-19-related work as an oral or poster presentation. The survey comprised eight items (Supplementary File 1), prompting respondents (hereafter referred to as “informants”) to describe changes to addictions research, policy and practice that occurred in their country of residence during the pandemic. Informants (*n* = 20) worked as researchers (*n* = 11), practitioners (*n* = 7), and policymakers (*n* = 1), and had many years of experience working in the field. They were residents of the following countries, in order of magnitude: Greece (*n* = 5); Netherlands, Nigeria, Poland (*n* = 2 each); Belgium, Canada, Egypt, Germany, Ireland, Italy, Jordan, North Macedonia, and the United Kingdom (*n* = 1 each) (see Supplementary File 2 for more details).

We used survey responses and our own experiences, complemented by peer-reviewed academic literature, to provide key examples of changes to practice, policy, and research. In the following sections, we present three case studies, focusing on telehealth, medication treatment for opioid use disorder, and alcohol harm reduction. These are followed by reflections on research changes and the ongoing implications of the pandemic for our collective work in the addictions field.

### Case study 1: telehealth as a response to COVID-19

Telehealth has been used in the addictions field for the last 20+ years, with some success, although limitations regarding interventions and research studies have been noted (6). Telehealth offered an opportunity to reduce disruptions to substance use disorder treatment services during COVID-19. Although various forms of telehealth have long been available in healthcare systems across many parts of the world, services for people with substance use disorders tend to be more rigid and less adaptable. The pandemic resulted in changes to such services, allowing more flexibility regarding how care and support were delivered. At the start of the pandemic, guidelines were changed to allow prescribers in Ireland to integrate telehealth to streamline access to medications for opioid use disorder (7) and, in the United States (US), allowed prescribers to initiate people onto buprenorphine without an in-person appointment (8). An informant from Poland highlighted that telehealth may be more difficult to integrate in some countries in Europe because of high variation of patient readiness (9). This was also the case in Greece, according to one informant, who reported that providers of substance use disorder treatment services did not recognize the possibilities for telehealth during COVID-19 and instead depended on phone calls and less advanced technology. A service in Greece which provided online therapeutic sessions for drug use reported that 45% of respondents found the service good or helpful, indicating areas for improvement (10). Another informant in Greece was concerned about the implications for data protection when providers continued using unencrypted instant messaging applications in services working with individuals whose behaviors are highly stigmatized and criminalized. In Nigeria, an informant reported that the service with the greatest impact during COVID-19 was DrugHelpNet (11), a network which disseminates phone numbers for frontline substance use clinicians to individuals in need of support.

Since the onset of COVID-19, the integration of telehealth into services has gone from incremental to cascading. However, treatment providers in Norway highlighted that important information (e.g., non-verbal/visual cues, smell) about patient wellbeing was not available via telehealth, suggesting that telehealth could serve as adjunct to face-to-face treatment, but not as a replacement (12). The rapid roll-out of telehealth also risks perpetuating health inequities by not being responsive to the needs of people who are linguistically diverse, unhoused, have little or no access to digital technology, or have multiple comorbidities. By implementing strategies which center the needs of these patient groups, telehealth offers the possibility of improving equity in health outcomes. Future iterations of telehealth which serve people experiencing substance use disorders should support low threshold access to care, while ensuring the security and confidentiality of those using the services.

### Case study 2: changes in the provision of medications for opioid use disorder

The use of medications for opioid use disorder is not new, with evidence that retention in such treatment reduces risk for all cause and overdose mortality (13). However, significant changes to provision of medication were made during the pandemic, some of which were seen as novel and innovative. For patients receiving daily medication treatment (e.g., buprenorphine, methadone) for opioid use disorder, treatment guidelines were relaxed in several countries to reduce in-person visits at treatment sites by allowing for longer take-home dosing intervals, typically comprising up to 14 days of medication (14, 15). In other countries (e.g., Canada, Norway), medication delivery to the patient's fixed home or temporary accommodation (e.g., shelter, COVID-19 isolation units) was also made possible, particularly in case of patients' COVID-19 infection (12). As a result of these adjustments to medication dispensing, the requirements for supervised dosing and saliva or urine drug screens were also decreased.

The rapid shift in guidelines focused on the maximization of available resources to prioritize the maintenance of treatment provision to existing and new patients, including rapid treatment induction, as mentioned by an informant from Ireland. In parallel, a scaling down of usual safety and surveillance measures (i.e., supervised dosing, drug screens) took place. For people engaged in treatment, reduced monitoring and increased flexibilities can improve quality of life (16). While the reduction of safety measures was welcomed by service user advocates, it also raised concerns around implications for patients themselves as well as the wider community (17). Unsupervised dosing can increase the risk of diversion and overdose (18, 19), although a systematic review concluded that there is “uncertainty about the effects of supervised dosing” (20). Treatment providers in Norway observed that flexible provision of take-home doses during COVID-19 led to time-savings, reduced treatment burden, and improved quality of life among patients (12). As an informant from North Macedonia noted, flexible medication provision also promoted retention in treatment. During the pandemic, the prescribing of depot buprenorphine subcutaneous injection as extend-release formulation alternative (i.e., weekly, or monthly dosing) to daily dosing was also scaled up in Australia, North America, and several European countries, offering increased convenience to patients (21). The long-term effects of the COVID-19 pandemic and changes to healthcare provision on morbidity and mortality among people who use opioids are still being assessed and will likely differ at national level. In the early stages of the pandemic, at least 25 countries had reported supply shortages of methadone and buprenorphine (22).

In North America, increasing prevalence of potent synthetic opioids in the drug supply has put people who use illicit opioids at increased risk of overdose (23). In response, guidelines in the Canadian province of British Columbia were amended to allow for safer supply interventions (e.g., prescribing of hydromorphone, psychostimulants) as a harm reduction strategy (24, 25). In the US, a record high in opioid deaths was reported for 2020 (26, 27) due to the toxic drug supply and has been linked to pandemic-related increases in patients' stress levels, social isolation, and polysubstance use as well as the above-described changes to opioid treatment provision (28). In England, deaths related to methadone (but not buprenorphine) went up by 64% in the first wave of the COVID-19 pandemic (March-June 2020) (29). However, this increase occurred not in methadone patients themselves but individuals outside of treatment, raising the question of potential diversion.

### Case study 3: changes to alcohol harm reduction approaches

Alcohol policies are an evidence-based way of reducing harm (30). Alcohol control measures adopted by national governments during the pandemic ranged from a relaxation of policies, such as permitting home deliveries of alcoholic beverages, to restrictions of hours of sale and temporary total bans of alcohol sales (31). Such total bans on alcohol were seen in two countries following national lockdowns in the spring of 2020. In India, a strict lockdown between 25^th^ March and 3^rd^ May 2020 resulted in a temporary ban on alcohol sales during this period (32, 33), while alcohol was declared a non-essential good in South Africa and therefore banned during lockdown (34, 35). In other countries, however, alcohol was declared an essential good, making alcohol widely available during lockdown periods (36). Informants noted changes in their countries, with alcohol becoming cheaper in Germany due to the reduction of VAT including on alcoholic beverages, and sales increasing in Greece. Multiple studies reported increases in alcohol consumption among those who drank heavily pre-pandemic and those experiencing alcohol use disorders, including dependence (37–41), highlighting a need for additional support resources.

While abstinence-based treatments are typically the norm globally for responding to alcohol use disorders, the pandemic provided opportunities to scale up alcohol harm reduction approaches (42). Alcohol harm reduction is much more limited than harm reduction approaches for drugs (43). Alcohol harm reduction was particularly important when rehabilitation and detoxification services closed completely or reduced access (42, 44). Where harm reduction had long been the approach to illicit drugs, the pandemic period saw the introduction of approaches targeting people experiencing alcohol use disorders, including access to medications to manage withdrawal (45); guidance for healthcare providers (45); safer drinking advice (46); and the increased provision of Managed Alcohol Programs, a specific harm reduction intervention for those experiencing alcohol dependence and homelessness (47–50). Such changes built on existing trends but changed practice in several areas, particularly where abstinence-based approaches were the norm.

Polarization of alcohol consumption, with an increase seen among those most vulnerable, will result in a significantly increased health and economic burden. If drinking patterns do not revert to pre-COVID patterns, the disease burden will be far higher (51). These increases in alcohol harm and costs to society could be prevented as part of COVID-19 recovery planning. This will prevent avoidable ill-health and premature deaths, reduce the impact on the healthcare system, and save money. Evidence already exists on the health benefits and cost-effectiveness of various alcohol control policies, which can complement other ongoing policy agendas. They can offer return on investment, are low cost, or can generate revenue, contributing to the health, social and economic recovery from the pandemic.

### Research changes

In many countries, the start of the pandemic led to face-to-face research activities being paused or significantly altered in 2020 to limit risk of infection (52). Within addictions research, as well as among many other areas of healthcare, clinical trials became impossible to initiate or implement as temporary bans on non-essential (i.e., non-COVID-19) studies were introduced (52, 53). This decrease in the initiation of non-COVID-19 studies began to rebound in late 2020 (53), coinciding with the start of COVID-19 vaccination programs.

Still, the COVID-19 pandemic highlighted the necessity of conducting addictions research outside of the clinic or laboratory, in more naturalistic settings, and reaching populations who may face significant barriers when engaging in research–and stimulated debate on more remote methods of study (52). Limitations on face-to-face, *in situ* research facilitated novel data collection approaches, including large sample online surveys [e.g., University College London COVID-19 Social Study with *n* = 33,644 (54)] as well as a more extensive use of secondary and big data. One informant from Ireland indicated the potential utility that app-based self-reported symptom logging could have for research on substance use disorders, similar perhaps to the ZOE COVID Symptom Study App (55) which has registered over 4 million users and already generated 50 scientific papers.

Involving community and peer-led organizations in the design and implementation of studies became crucial not only to improve their impact, but also to strengthen the studies' feasibility and sustainability in the face of challenging conditions of the pandemic research environment. Patient and public involvement (PPI) or citizen science (science conducted with participation from the public) as well as open science offer the possibility of “engaged citizenship” (56, 57). Yet, the COVID-19 pandemic also drew attention to the limits of community-based participatory research, particularly power imbalances and inherent structural issues such as digital inequality that can sideline the target populations involved (58).

The working environment of addictions researchers also changed drastically during the pandemic, with office closures requiring many to work remotely for extended periods of time. Moreover, in some countries, the temporary halt to non-essential clinical research projects (see above) at the start of the pandemic led to research staff being furloughed (i.e., suspended for enforced period of work absence), for instance under the United Kingdom Coronavirus Job Retention Scheme (59). Further, for addictions researchers working in public health agencies, some staff were temporarily transferred to special COVID-19 units to support pandemic emergency response efforts (60, 61). For those who remained in employment, remote working facilitated collaboration between practitioners and researchers due to the ease of online meetings, as one informant from the Netherlands remarked. Initiatives such as the SU x COVID Data Collaborative were also agile responses to the pandemic, bringing together scientists and community health practitioners to promote data collection internationally (62).

Given the emergency nature of the pandemic, new funding opportunities were rapidly put in place to facilitate timely research. The urgency of the crisis and need for timely public health responses impacted research designs, and rapid assessments were frequently undertaken to inform policy, as “*cost-effective and pragmatic research […], particularly when inadequate data exist”* (63) [see also (64)]. An informant from Canada noted the continued need for creative approaches to pitching ideas for addictions research as the response for these COVID-19 funding calls. Funding and collaboration opportunities also emerged in low- and middle-income countries, where ordinarily funding for addictions research has been scarce, including Albania, Jordan, and Nigeria. A Nigerian informant highlighted: “During [the] COVID-19 pandemic, government agencies and major stakeholders became more proactive, intervention programs provided new insights on how best to handle addictions in emergency situations coupled with evidence-based, result-oriented approaches both in policy, practice and research.”

## Discussion

The poly-crisis of the COVID-19 pandemic and substance use disorders has pushed at the boundaries of research, practice and policy but also offered opportunities for the future, particularly regarding collaboration and community engagement within the addictions field. Equity should, however, be centered in research and funding going forward to ensure benefits of research are equally distributed, as much of the learning has been from those currently engaged in treatment in high-income countries. Building on examples of good practice in research methods established during the pandemic, as well as setting compensation for participation, allows for the involvement of people who might otherwise be excluded.

The same applies to collaboration within academia, which has benefited enormously from the online environment, enabling international research collaborations. Eventually, the work of community-based organizations in research over the last 3 years and beyond needs to be recognized to create sustainable structures promoting collaboration between community, practice, and research.

The treatment systems which proved to be most resilient during the pandemic were likely those with sufficient resources to embrace innovations in drug development (such as depot buprenorphine) and technology (telehealth) and meet the needs of people experiencing substance use disorders by tailoring treatment and harm reduction services to their current living situations.

It is important to note that this piece largely draws on the experiences of practitioners in high-income countries, and additional funding for innovations and further research will be required to understand the impact of the same interventions in low- and middle-income countries.

The COVID-19 pandemic was one crisis, which had considerable impact on the addictions field. However, there are lessons to be learned to ensure drug and alcohol services and related research is sustainable globally, in ongoing and future crises, in terms of emergency preparedness. Climate change and the war in Ukraine (65, 66) are two such examples where such lessons can be applied. From our experience and the information gathered as part of our working group, we suggest the following implications for policy, practice, and research:

- Telehealth services offer the possibility of providing more flexible care, but they should only be offered in circumstances where data systems are secure enough to handle confidential client information;
- Scale-up of telehealth risks excluding people who are linguistically diverse, unhoused, have little or no access to technology, or have multiple comorbidities. Implementation strategies should prioritize equity in access to such technology;
- The pandemic period saw a scale-up of prescribed safer supply. This “policy window” could be taken advantage of in countries where safe supply is not already available;
- Flexibility in the provision of medication for opioid use disorder should be sustained to allow services to respond to the needs of their clients whilst also ensuring risks are minimized;
- The pandemic period demonstrated the possibilities for integrating harm reduction approaches into services for alcohol use disorders. These experiences should be built upon to inform scale-up;
- The limits on drug and alcohol research during the COVID-19 pandemic also provoked creative and international collaborations. Sustainable research should center the voice of people experiencing substance use disorders;
- The pandemic period saw the roll-out of novel approaches in drug and alcohol services, often based on imperfect knowledge. Research and funding bodies must consider how they can support changing knowledges, particularly in low- and middle-income countries, where such innovations may be limited due to a lack of funding.

## Conclusion

The COVID-19 pandemic drove a poly-crisis which challenged addiction practice, research and policy. Now, 3 years from the onset of the pandemic, we have witnessed various transformations in the addiction field, including many positive ones, from which we can and should learn for the future to be prepared for ongoing and emerging crises.

## Data availability statement

The datasets presented in this article are not readily available because the survey dataset described in this paper is not publicly available. Requests to access the datasets should be directed to HC, hannah.carver@stir.ac.uk.

## Author contributions

HC and TC co-facilitated the InterGLAM working group. All authors contributed to the writing and editing of the final manuscript.
