# Supplementary material for: Substance use disorders and COVID-19: reflections on international research and practice changes during the “poly-crisis”
Source: Front Public Health. 2023 Jul 17;11:1201967. doi: 10.3389/fpubh.2023.1201967 (PMC10390069; doi:10.3389/fpubh.2023.1201967)
Supplement: Supplementary file 1 [file Data_Sheet_1.pdf]

InterGLAM working group 5

Page 1: Introduction to the survey

This survey has been created by members of the InterGLAM working group 5 who are looking at the changes to addictions policy, practice and research as a result of the COVID-19 pandemic. We would like to gather examples of these changes globally, so if you have examples of changes to policy, practice and/or research in relation to problem drug and/or alcohol use, please complete the survey. We will be using the information provided (anonymously) in a commentary paper.

Page 2: Questions

1. Which option best describes your role? (Choose the most appropriate answer)

- Researcher/academic
- Practitioner
- Policy maker

2. Which country do you live in or are most familiar with (i.e., if you live in one country but work/do research in another)?

3. Please tell us about a positive change that has been made to policy, practice or research in your country as a result of the COVID-19 pandemic. This should be relevant to the role you chose above (i.e., if you are an academic, please provide information about research changes).

4. If information about this change is in the public domain/has been published, please provide more information/any weblinks.

5. Please tell us about any unintended negative consequences of the pandemic in your country.

6. Please tell us about any changes to funding for research/policy/practice changed in your country during the pandemic.

7. What are the key lessons learned from the pandemic that have resulted in improvements for people who use drugs/alcohol in your field and/or country?

8. What are the key future research questions that need to be addressed following the changes that have been observed during the pandemic?

9. We would like to contact you for more information. If you are happy for a member of the working group to contact you, please provide your email address.

Page 3: Final page

Thank you for participating. If you have any questions please contact the working group 5 co-facilitators: Dr Hannah Carver (hannah.carver@stir.ac.uk) or Dr Teodora Ciolompea (teodora.ciolompea@gmail.com) or the InterGLAM team (INTER-GLAM@clinic.cat).
