# Supplementary material for: Substance use disorders and COVID-19: reflections on international research and practice changes during the “poly-crisis”
Source: Front Public Health. 2023 Jul 17;11:1201967. doi: 10.3389/fpubh.2023.1201967 (PMC10390069; doi:10.3389/fpubh.2023.1201967)
Supplement: Supplementary file 2 [file Data_Sheet_2.docx]

**Supplementary File 2**

**
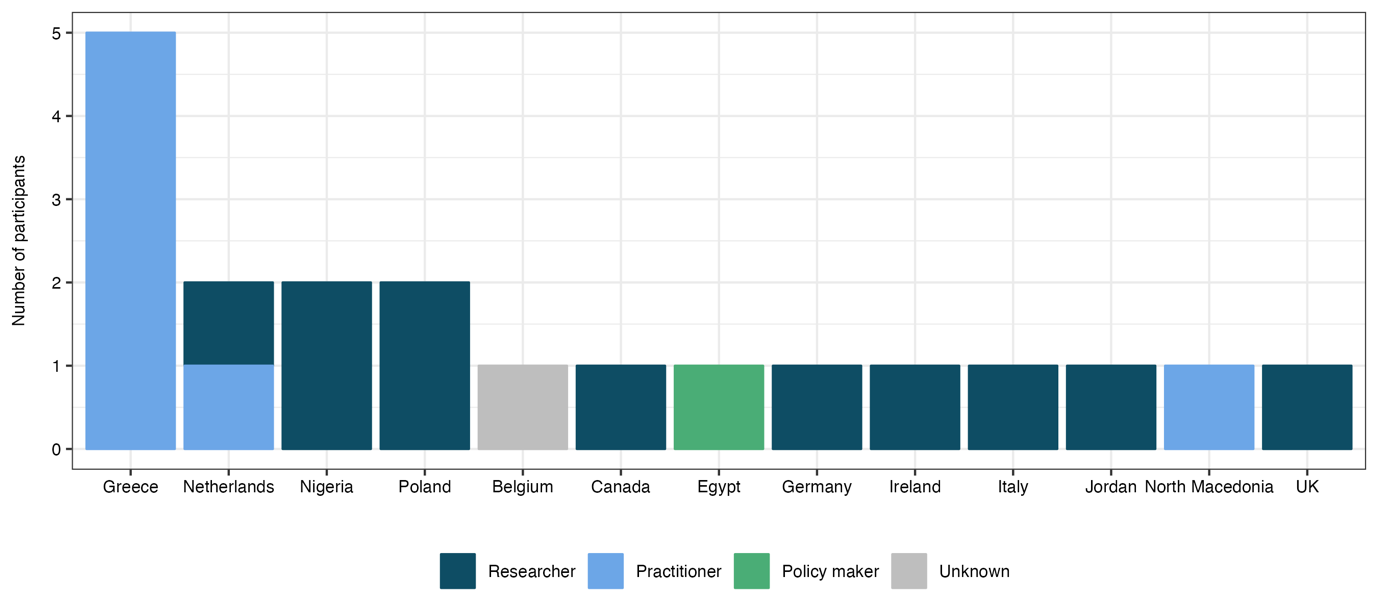
**

Figure S1. Survey participants by country and role. Total number of participants: n = 20.

Note. Survey question 1: In the addictions field, which option best describes your role? Survey question 3: Which country do you live in or are most familiar with (i.e., if you live in one country but work/do research in another)?

**
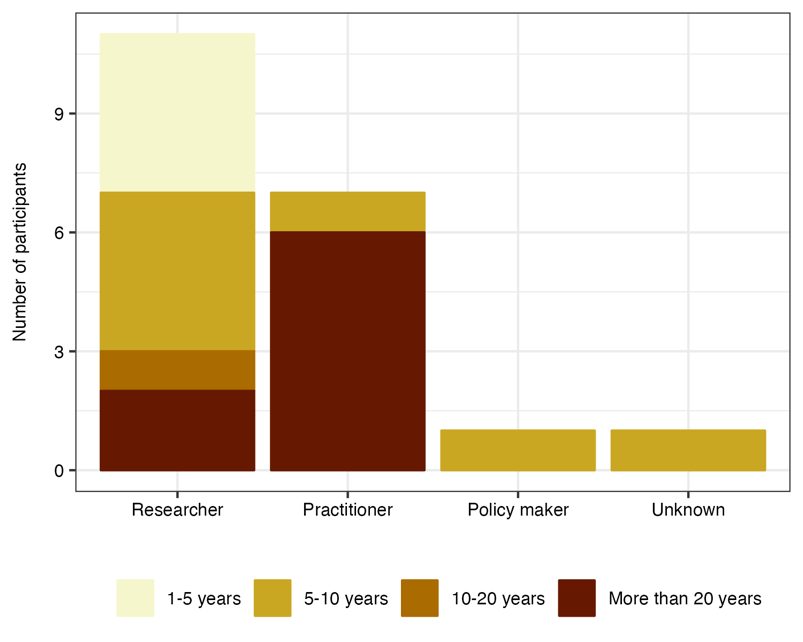
**

Figure S2. Survey participants by role and years of experience in the addiction research field. Total number of participants: n = 20.

Note. Survey question 1: In the addictions field, which option best describes your role? Survey question 2: How long have you been working in the addictions field?
